# Chronic extradural compression of spinal cord leads to syringomyelia in rat model

**DOI:** 10.1186/s12987-020-00213-4

**Published:** 2020-07-31

**Authors:** Longbing Ma, Qingyu Yao, Can Zhang, Mo Li, Lei Cheng, Fengzeng Jian

**Affiliations:** 1 Department of Neurosurgery, China International Neuroscience Institute, Xuanwu Hospital, Capital Medical University, 45 Changchu Street, Beijing, 100053 China; 2 Cell Therapy Center, Xuanwu Hospital, Capital Medical University, Beijing, China; 3 Research Center of Spine and Spinal Cord, Beijing Institute for Brain Disorders, Capital Medical University, Beijing, China

**Keywords:** Central canal, CSF, Extradural compression, Rat model, Syringomyelia

## Abstract

**Background:**

Syringomyelia is a common spinal cord lesion. However, whether CSF blockage is linked to the formation and enlargement of syringomyelia is still controversial. The current model of syringomyelia needs modification to more closely mimic the clinical situation.

**Methods:**

We placed cotton strips under the T13 lamina of 40 8-week-old rats and blocked CSF flow by extradural compression. After 4 and 8 weeks, MRI was performed to evaluate the morphology of syringomyelia and the ratio of spinal cord diameter to syrinx diameter calculated. Locomotor function was evaluated weekly. Spinal cord sections, staining and immunohistochemistry were performed 8 weeks after surgery, the ratio of the central canal to the spinal cord area was calculated, and ependymal cells were counted. In another experiment, we performed decompression surgery for 8 rats with induced syringomyelia at the 8th week after surgery. During the surgery, the cotton strip was completely removed without damaging the dura mater. Then, the rats received MRI imaging during the following weeks and were sacrificed for pathological examination at the end of the experiment.

**Results:**

Syringomyelia formed in 82.5% (33/40) of rats at the 8-week follow-up. The Basso, Beattie and Bresnahan (BBB) scores of rats in the experimental group decreased from 21.0±0.0 to 18.0 ±3.9 in the first week after operation but returned to normal in later weeks. The BBB score indicated that the locomotor deficit caused by compression is temporary and can spontaneously recover. MRI showed that the syrinx is located in the center of the spinal cord, which is very similar to the most common syringomyelia in humans. The ratio of the central canal to the spinal cord area reached (2.9 ± 2.0) × 10^−2^, while that of the sham group was (5.4 ± 1.5) × 10^−4^. The number of ependymal cells lining the central canal was significantly increased (101.9 ±  39.6 vs 54.5 ± 3.4). There was no syrinx or proliferative inflammatory cells in the spinal cord parenchyma. After decompression, the syringomyelia size decreased in 50% (4/8) of the rats and increased in another 50% (4/8).

**Conclusion:**

Extradural blockade of CSF flow can induce syringomyelia in rats. Temporary locomotor deficit occurred in some rats. This reproducible rat model of syringomyelia, which mimics syringomyelia in humans, can provide a good model for the study of disease mechanisms and therapies.

## Background

Syringomyelia refers to cystic dilatation in the spinal cord, usually secondary to various diseases, such as Chiari malformation, spinal cord injury, tumor, spinal arachnoiditis, tethered cord, etc [1–4]. Despite the extensive use of imaging studies, animal experiments and clinical studies, the pathophysiological mechanism for syringomyelia is still unclear [5–7]. Some studies have shown that the formation of syringomyelia may be closely related to obstruction of CSF flow [8, 9]. Clinically, after the factors causing syringomyelia are removed and CSF circulation is restored, the prognosis of patients is variable, and the syringomyelia of many patients does not resolve [10, 11]. This phenomenon is unclear and requires further study.

To study a disease, researchers must establish a reproducible animal model that displays high similarity to human disease. Previous methods for establishment of a model of syringomyelia include injecting excitotoxic acid into the spinal cord parenchyma [9, 12] or kaolin into the subarachnoid space [13] and cisterna magna [6, 14–19], or inducing spinal cord injury by a spinal cord impactor [20]. Kaolin can spread in the vertebral canal and cause wide intraspinal inflammation. But the induction with kaolin is irreversible, the damage to spinal cord is persistent, and it is impossible to observe any recovery process. Therefore, we need a reversible syringomyelia model to demonstrate whether recovery from syringomyelia can occur after surgical treatment. In addition, previous models have been limited by difficult operations and a high incidence of complications. These factors have limited the experimental study of the molecular mechanisms and therapeutics for syringomyelia. Previous models have used both canalicular [13, 16, 21, 22] and noncanalicular [23] syringomyelia to mimic different causes. Most syringomyelia in humans is located in the central canal. In our study, extradural compression was used to obstruct CSF flow and duplicate canalicular syringomyelia in rats.

## Methods

### Experiment 1

### Animals and surgical procedures

**Fig. 1:**
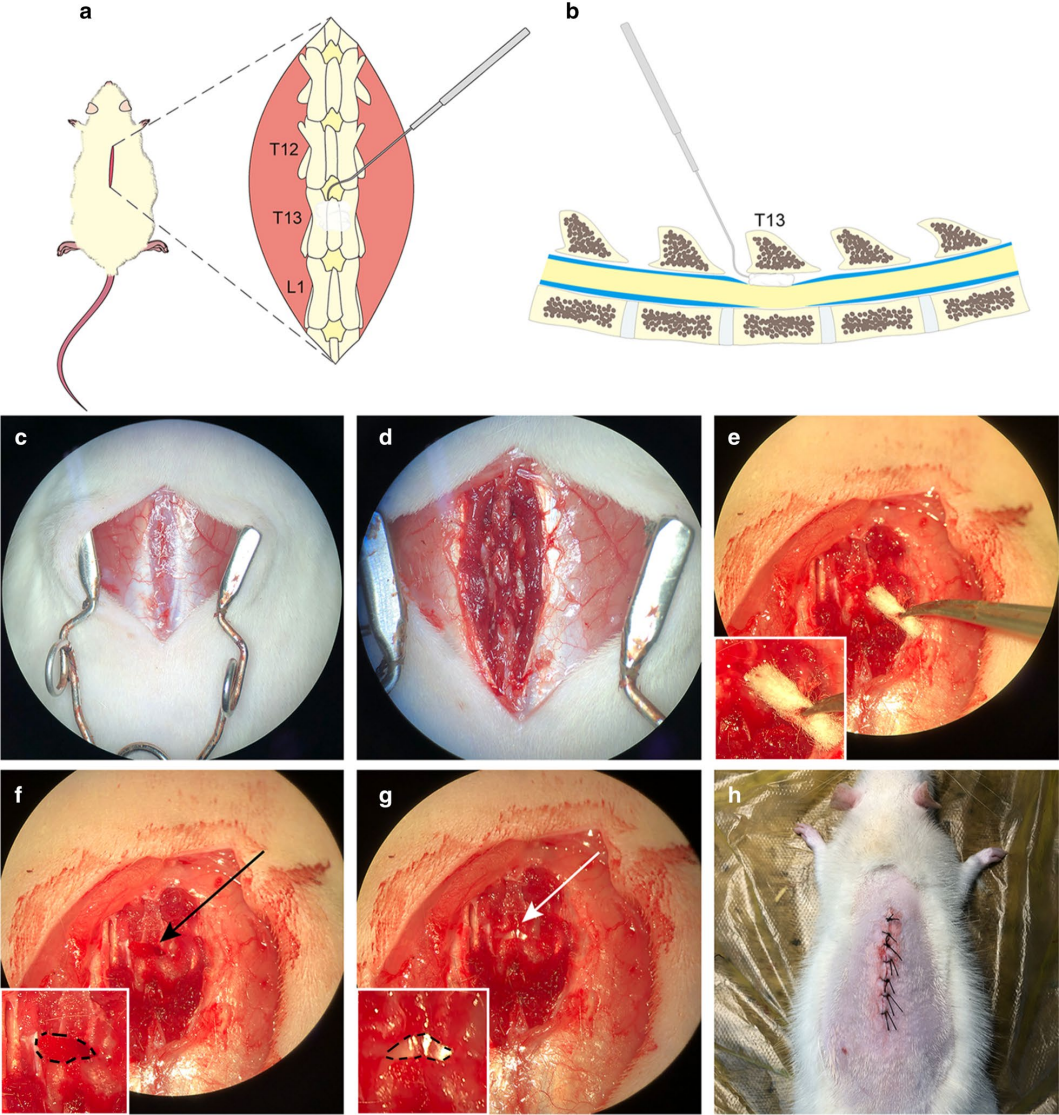
Schematic and intraoperative photos of the procedure to induce syringomyelia. **a** Dorsal view and **b** lateral view: a cotton strip was stuffed under the T13 lamina from the T12–13 laminae interval space by bending the nerve stripper. **c** Procedure: A 3 cm long incision was made in the middle of the rat’s back. **d** T12–13 lamina were exposed. **e** The ligamentum flavum was cut open with micro-scissors. Fifteen milligrams of cotton were weighed and molded into cotton strips. **f** After the cotton strip (*black arrow and area marked with black dotted lines*) was added, the dura mater was directly attached to the spinal cord, and CSF flow was blocked (**g**, *white arrow; The black dotted line outlines the interlaminal space*). **h** The incision was sutured

A total of 50 female 8-week-old Sprague–Dawley rats (WeiTongLiHua Corp., Beijing, China) were used in this study. The body weight of the rats ranged from 230 to 280 g. The experimental group consisted of 40 rats, while the sham (control) group included 10 rats. The experimental group received extradural compression (Fig. 1a, b), while the control group were sham operated. The animal experiments were approved by the Animal Ethics Committee of our institution and conformed to China Animal Management Regulations. Rats were bred under standard housing conditions at the Animal Experiment Center of Xuanwu Hospital.

Enflurane (Yipin Corp., Hebei, China), nitrous oxide and oxygen mixed gas were used for general anesthesia. Anesthesia induction was performed in an anesthesia chamber at a dose of 2% enflurane in 70% nitrous oxide and 30% oxygen. A rat respiratory mask was used to maintain anesthesia during the operation with a dose of 1.0–2.5% enflurane in 70% nitrous oxide and 30% oxygen (Bickford veterinary anesthesia equipment model no. 61010; AM Bickford, Inc., Wales Center, NY, USA). The rats were placed in the prone position with their limbs fixed on a thermostatic operating bed at a temperature of 36 °C. After the hair on the back was shaved, 1% iodophor solution was used for skin sterilization. A skin incision was made along the middle of the back with T13 as the center, with a length of approximately 3 cm (Fig. 1c). Subcutaneous tissue was carefully separated, muscle tissue was stripped from the lamina, and the T12, T13 and L1 vertebral laminae were completely exposed (Fig. 1d). The T12–13 laminae interval space was carefully separated, exposing the ligamentum flavum. Then, the T13 spinous process was gently lifted with a hemostat, and the ligamentum flavum was gently cut along the middle position of the T12–13 intervertebral space with micro-scissors. After these steps, the transparent dura mater was exposed, and CSF under the dura mater was observed. Aseptic absorbent cotton balls (Chuangxin Corp., Shandong, China) were weighed using an electronic analytical balance (measuring range: 1 mg-120 g, LiChen Corp., Hunan, China). These cotton balls are common absorbent cotton balls widely used in hospitals and laboratories and are not limited to a specific manufacturer, were shaped into a long strip by hand and placed under the T13 spinous process (Fig. 1e). Cotton strips weighing 15 mg were gently stuffed into the extradural space below the T13 lamina from the incision of the ligamentum flavum in the T12–13 lamina space with a homemade bending nerve stripper (Fig. 1f). We ensured that the metal stripper did not directly contact the dura mater because the dura mater is very thin and extremely fragile. During the procedure, the dura mater must remain intact. After the cotton strip was completely placed, the compressed dura mater was observed to be on the surface of the spinal cord, and the CSF space was obstructed (Fig. 1g). After this the region was flushed with saline, the muscles and skin were sutured and closed. Ten rats in the sham group were given a sham operation, which also exposed the T12–13 interlaminar space and dura mater but did not involve cotton strips. All operations were performed according to the principle of aseptic operation and under a surgical microscope (OPMI Pico, Carl Zeiss, Oberko Chen, Germany) using a magnification of 16 times or 25 times. Intraperitoneal cefuroxime was administered for 2 days after the operation to prevent infection. All rats were kept and observed in conventional and clean rat houses.

### Behavioral testing

For all rats in the experimental group and the sham group, locomotor functions were evaluated weekly after operation until sacrifice. We used Basso, Beattie and Bresnahan (BBB) scoring of open field walking to evaluate the hind limb locomotor function of the rats [24].

### In vivo MRI

All rats (experimental group: n = 40 and sham group: n = 10) underwent serial MRI scans 4 and 8 weeks after the operation.

In vivo MRI was performed using a 7.0 Teslan MRI scanner (PharmaScan 7T, Bruker Corp., Karlsruhe, Germany) with 400 mT/m gradients in the Animal Imaging Experimental Center at Capital Medical University. The rats were placed prone on the table with two restraining belts to fix the trunk. General anesthesia was induced by 4% isoflurane in oxygen before scanning and maintained by 2% isoflurane in oxygen via a rat mask during scanning. The body temperature, heart rate and respiration were closely monitored during imaging.

After we performed rapid whole-body localization scans in all three planes, sagittal and axial T2 weighted images were acquired with the operation area as the center by using a fat-saturated RARE sequence. A rat volume coil with a diameter of 89 mm was used for transmission and to obtain data. Imaging parameters for sagittal acquisition were TR/TE = 3000/33 ms, matrix size = 256 × 256, field of view (FOV) = 60 × 40 mm^2^, slice thickness = 600 μm with no gap, number of slices = 10, NEX = 8, and resolution = 0.147 × 0.147 × 1 mm^3^. The imaging parameters for axial acquisition were TR/TE = 4500/33 ms, matrix size = 256 × 256, FOV = 60 × 40 mm^2^, slice thickness = 1 mm with no gap, number of slices = 30, NEX = 8, and resolution = 0.147 × 0.147 × 1 mm^3^. Each MRI scan took approximately 12 min.

The anteroposterior diameter of the syrinx every 1 mm from the full length of the syrinx was measured in sagittal T2-MRI images. And the largest diameter among the above data was selected and the AP diameters of the spinal cord in the same plane were measured to calculate the ratio. All measurements were made using the Horos software platform (v3.3.5, https://horosproject.org).

### Histological analysis

Eight weeks after the operation, all rats in both groups were euthanized by an overdose of pentobarbital sodium (150 mg/kg IP). They were perfused with 4% paraformaldehyde in 0.01 mol/L PBS, and the whole spinal column was removed and fixed in 4% paraformaldehyde for 24 h. Then, the spinal cord (approximately 6 cm long and centered on the operation site) of the operation segment was harvested carefully to maintain its integrity. After dehydration, the spinal cord was embedded in melted paraffin and then sectioned at a thickness of 3 μm in the axial plane. Sections were stained with hematoxylin and eosin (HE), Luxol fast blue (LFB) and Nissl under standard procedures. On HE-stained sections, the areas of the central canal and spinal cord were measured at the maximal diameter of the syrinx and the ratio of the central canal to the spinal cord area was calculated to evaluate the size of the syrinx. The number of ependymal cells in the two groups was also counted manually on the transverse sections. Three slices were selected for counting for each rat, namely the largest part of syringomyelia and the 5 mm plane above and below it. All stained sections were scanned with a high-resolution pathological section scanner (Panoramic MIDI, 3DHISTECH, Hungary). All measurements were made in ImageJ with FIJI installed (NIH, https://imagej.nih.gov/ij/).

### Immunohistochemistry

Immunohistochemistry was carried out in both the experimental group and the sham group rats. Sections were deparaffinized in xylene for 3 changes and sequentially incubated in pure ethanol and graded ethanol solution for dehydration. Slides were immersed in sodium citrate antigen retrieval solution (pH 6.0) and maintained at a sub-boiling temperature to retrieve antigen. Sections were immersed in 3% H_2_O_2_ to block endogenous peroxidase and blocked with 3% BSA at room temperature for 30 min. Slides were incubated with primary antibodies (diluted with 3% BSA appropriately) overnight at 4 °C. The primary antibodies used were rabbit anti-IBA1 (Abcam, ab178847, 1:8000) and rabbit anti-MBP (CST, 78896T, 1:200). The secondary antibody (HRP labeled) corresponding to the primary antibody was added to cover the tissue and incubated at room temperature for 50 min. Sections were stained with DAB chromogenic reagent, stained with hematoxylin, dehydrated, and sealed. The average optical density (AOD) of the two groups of sections was calculated and compared. The formula AOD= IOD/area was used for AOD calculation (integrated optical density, IOD). All sections were scanned using a high-resolution pathological section scanner (Panoramic MIDI, 3DHISTECH, Hungary) and analyzed using ImageJ with FIJI installed (NIH, https://imagej.nih.gov/ij/).

### Experiment 2

#### Decompression operation

**Fig. 2:**
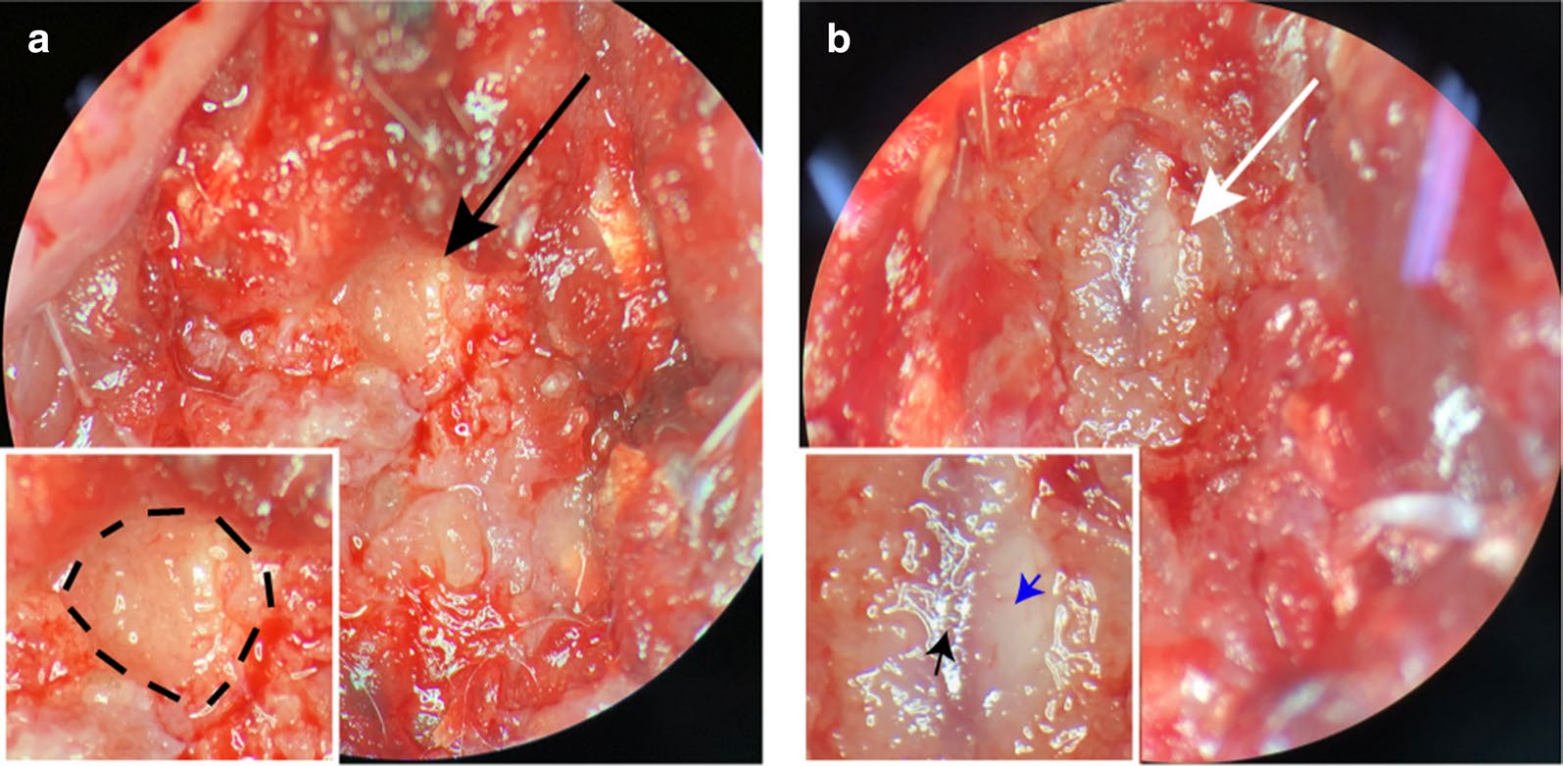
Photos of the decompression operation. **a** After the T13 lamina was ground off with a high-speed microdrill, the cotton strip (black arrow and area surrounded by black dotted lines in the magnified picture) was completely exposed. **b** The cotton strip was carefully removed with microscopic tweezers. Because the dura mater is very weak, care was taken to prevent rupture of the dura mater during the removal. After the cotton strip was completely removed, CSF was seen to refill the subarachnoid space under the transparent dura mater (*white arrow*). The blue arrow refers to the transparent dura mater. The black arrow refers to blood vessels on the surface of spinal cord. All procedures were performed under a ×16 surgical microscope

We induced syringomyelia in another 8 rats using the same method as above and then tried to reverse this operation 8 weeks after the first operation. The presence of syringomyelia was confirmed on MRI in all rats. The procedure was as follows: anesthesia, disinfection and position placement were performed as described above, and then, the skin, fascia and muscle of the first operation site were cut to expose the T13 lamina. The T13 lamina was carefully ground with a high-speed microdrill. After the cotton strip was completely exposed (Fig. 2a), it was carefully cut off using micro-tweezers and micro-scissors. In this way, the T13 lamina and cotton were removed, creating enough space to restore the CSF flow. CSF refilling the subarachnoid space was observed under the transparent dura mater, indicating successful decompression (Fig. 2b). Four and eight weeks after decompression, MRI scanning was performed to observe spinal cord decompression and changes in syringomyelia. Eight weeks after the decompression operation, all 8 rats were sacrificed to obtain the spinal cord and perform histological evaluation.

### Statistical analysis

All of the experimental values are expressed as the mean ± SD. BBB scores and the ratio of the central canal to the spinal cord diameter between the experimental and sham groups were compared using two-way RM ANOVA followed by a post hoc test using the Tukey test. The Mann-Whitney test was used for the intergroup comparison of the ratio of the central canal to the spinal cord area and the number of ependymal cells. Additionally, comparisons of MBP and IBA1 expression between the control and experimental animals were performed using unpaired t tests. All quantification was performed blindly by assigning a code letter to each rat and deciphering this code only after all data analyses were complete. Statistical evaluations were conducted using GraphPad Prism 6 (GraphPad Software, San Diego, USA), and a *P* value <0.05 was considered statistically significant.

## Results

A total of 40 rats underwent an operation to induce syringomyelia. Cotton strips weighing 15 mg were stuffed under the T13 lamina to compress the spinal cord from the extradural space and block CSF flow. Ten rats underwent a sham operation. No rats died during or after the operation.

**Fig. 3:**
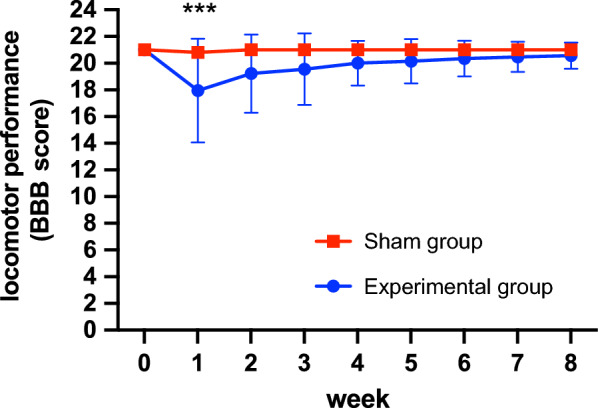
BBB locomotor scores of the experimental group and sham groups weekly after induction of syringomyelia. Two-way RM ANOVA followed by a post hoc test using the Tukey test was used for intergroup comparisons. During the follow-up period of 8 weeks, the BBB score of the rats in the sham group did not change significantly, but in the first week, the locomotor function of the rats in the experimental group decreased significantly (BBB score decreased from 21.00 ± 0.00 to 17.95 ± 3.88), and there was a significant difference between the two groups. However, from the second week to the 8 week, the motor function of the rats in the experimental group gradually improved, and there was no significant difference from the sham group from the second week. This result shows that the motor function of the experimental group could recover spontaneously without treatment. ***P < 0.001

BBB locomotor scores were determined in all experimental group and sham group rats to evaluate motor function. The BBB scores of the rats in the experimental group decreased significantly from 21.0 ± 0.0 to 18.0 ± 3.9 in the first week after the operation, while the rats in the sham operation group only showed a decrease to 20.8 ± 0.4, with a significant difference between the two groups. These results indicate that the compression caused by the operation, rather than an operation only, causes certain damage to the spinal cord, causing a decrease in locomotor function. However, the BBB scores in the experimental group increased significantly at 2 and 3 weeks after operation, reaching 19.2 ± 2.9 and 19.6 ± 2.7, respectively, and finally recovered to 20.6 ± 1.0 at 8 weeks. From the second week onwards, there was no significant difference between the experimental group and the sham operation group. At the end of 8 weeks, the lower limb motor function of the rats in the experimental group returned to normal, and only 2 rats had residual unilateral motor dysfunction. These results showed that the spinal cord injury caused by compression was temporary and could recover spontaneously (Fig. 3).

**Fig. 4:**
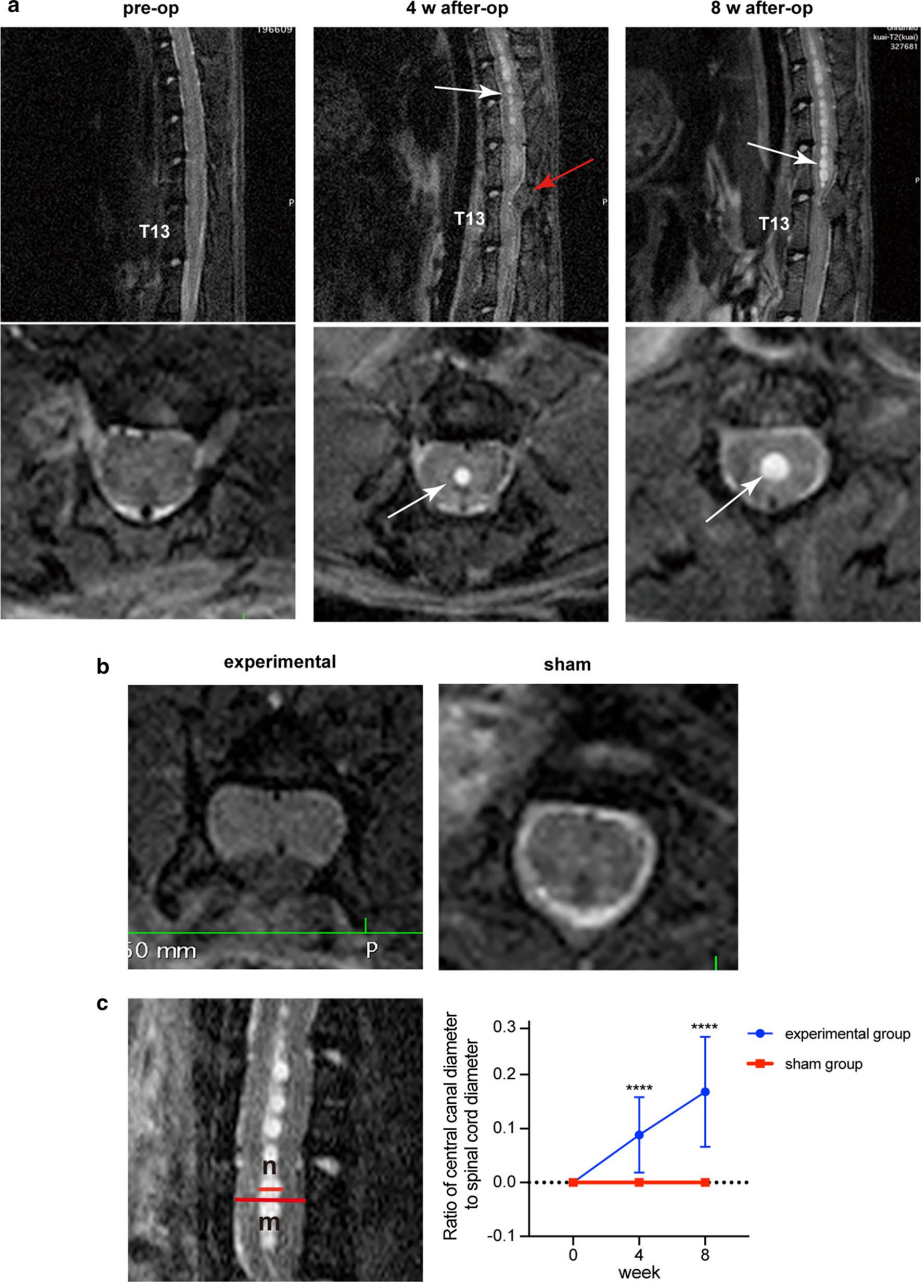
Serial T2-weighted MRIs of rats. **a** MRI shows the process of central canal dilatation and syringomyelia enlargement. Four weeks after the operation, Sagittal and axial T2-weighted MRI showed the cotton strip was added (red arrow), and syringomyelia enlarged gradually (white arrow). At 8 weeks, syringomyelia was significantly larger than that of 4 weeks. **b** CSF signal disappears after cotton strip compression in experimental group, while CSF signals in the sham group were obvious. **c** n/m represents the ratio of the central canal diameter to the spinal cord diameter on MRI in the plane of the largest syrinx. Linear trend of n/m at 4 and 8 weeks. ****P < 0.0001

After extradural compression in the experimental group, 27 (67.5%) rats and 33 (82.5%) rats showed syringomyelia on T2-weighted MRI at 4 weeks and 8 weeks, respectively. The sham group did not show any syringomyelia after the sham operation. In all rats with syringomyelia, the syrinx was located rostral to the compression site, at approximately T10-12, with a length of 2–3 spinal segments. The syrinx was located in the center of the spinal cord and appeared bead like (Fig. 4a). On T2-weighted MRI, CSF flow was obstructed by cotton strips (Fig. 4b). After the size of the syrinx increased, the separation disappeared and merged into one longer syrinx. The ratio of the diameter of the central canal to the spinal cord in the experimental group by MRI was 0.088 ± 0.070 at 4 weeks and increased to 0.17 ± 0.10 at 8 weeks (Fig. 4c). There was a significant difference between the experimental group and the sham group at two time points (*P* < 0.0001). These results indicate that the syrinx gradually increased in size after the operation to induce syringomyelia. In our results, the largest syrinx reached 32% of the spinal cord diameter.

**Fig. 5:**
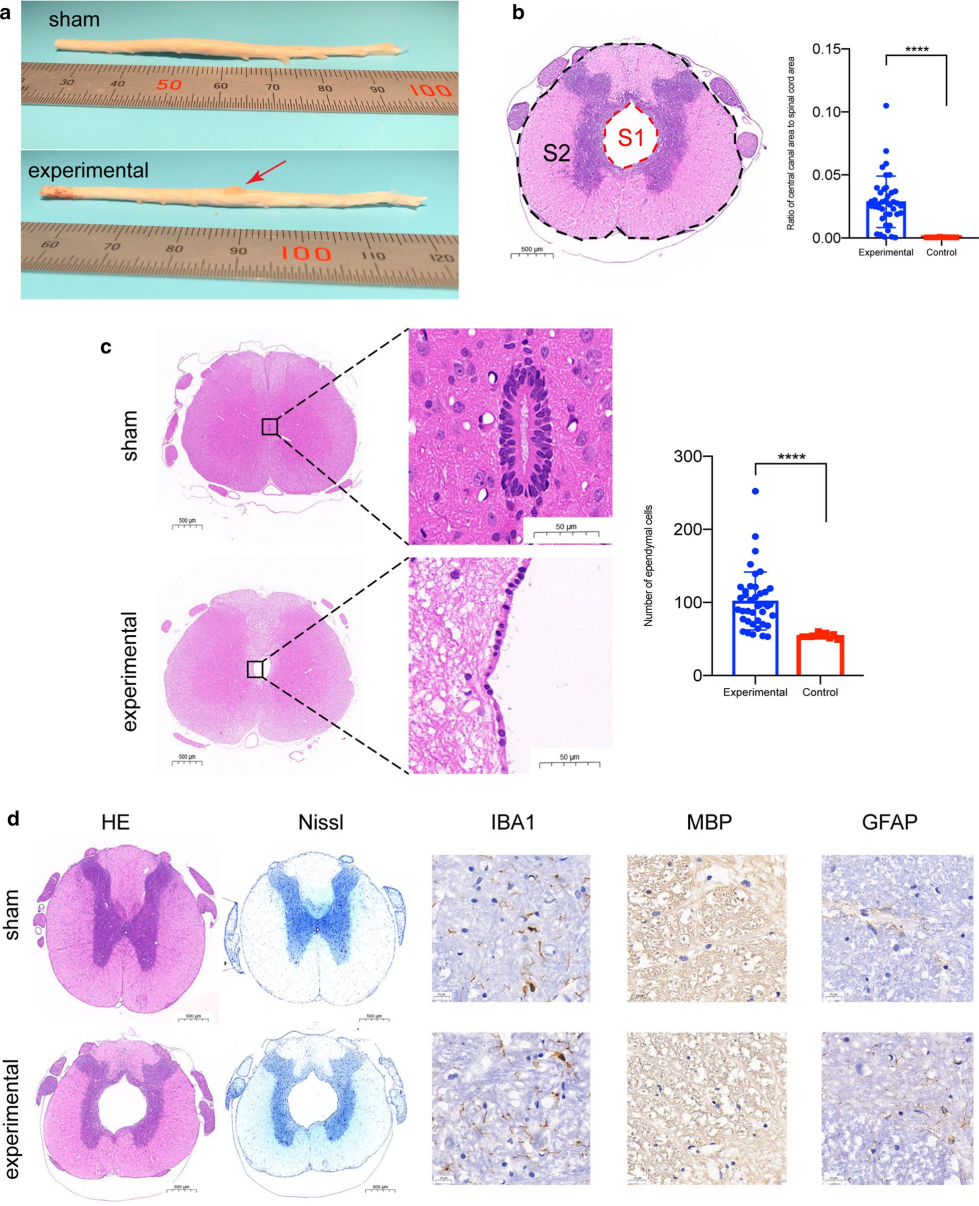
Histopathological and immunohistochemical evaluation of syringomyelia. **a** After fixation for 24 h, the spinal cord was carefully harvested. *The red arrow* indicates the cotton strip. **b** The ependyma of the central canal is outlined with red dashed lines, and the area is calculated to represent the cross-sectional area of the central canal (*S1*). The outer border of the spinal cord is outlined with black dashed lines, and the area is calculated to represent the cross-sectional area of the spinal cord (*S2*). The ratio of the central canal to the spinal cord area = *S1*/*S2*. **c** Local magnification of the central canal in the sham group (HE staining) shows that the ependymal cells are closely arranged around the central canal. The area of the central canal is very small, close to the recessive space. The ependymal cells lining the central canal of rats in the sham group were distributed in one layer, and the intercellular space was larger than that of the sham group. Ependymal cells were counted in the entire margin of the central canal. **d** At 8 weeks, the spinal cords of rats in both groups were removed and transected. Routine HE staining, Nissl staining, and IBA1 and MBP immunohistochemistry were performed. In the experimental group, the central canal of the spinal cord was obviously dilated, and the surrounding gray matter was pushed to all sides. There was no difference in IBA1 staining between the experimental group and the sham group. There was no difference in MBP staining between the experimental group and the sham group. ****P < 0.0001

Furthermore, we measured the ratio of the central canal to the spinal cord area in all rats at 8 weeks on histological sections to evaluate the size of the syrinx. The ratios were (2.9 ± 2.0) × 10^−2^ and (5.4 ± 1.5) × 10^−4^ in the experimental group and sham group, respectively (Fig. 5b). There was a significant difference between the two groups (*P* < 0.0001). This result indicates that the central canal in the experimental group is significantly expanded. In the 8-week syringomyelia induction experiment, the area of the central canal was increased 53 times. Of the 40 rats in the experimental group, only 4 rats had the central canal area expanded by less than fivefold. If expansion of the central canal area by fivefolds is defined as the criteria of syringomyelia, 90% of the rats had successfully induced syringomyelia, which is higher than the positive rate on MRI. On pathological sections, our models all showed syringomyelia formed by central canal dilatation. There were no vacuoles in the spinal cord parenchyma and no scar formation on the dura mater, arachnoid or spinal cord surface. The morphology of the spinal cord remained intact with an undamaged outer surface of the spinal cord and anteromedian and posteromedian grooves. The central canal with the ependymal cells lining it was not ruptured, but the gray matter of the spinal cord was displaced laterally by the enlarged central canal to varying degrees (Fig. 5c, d). There was no difference in IBA1 staining between the experimental group and the sham group, and there was no significant difference in the AOD value between the two groups (0.10 ± 0.01 vs 0.10 ± 0.01, *P* = 0.72). There was also no difference in MBP staining between the experimental group and the sham group, and there was no significant difference in the AOD value between the two groups (0.21 ± 0.01 vs 0.20 ± 0.01, *P* = 0.61). With the enlargement of the central canal, the arrangement of ependymal cells became detached, and the intercellular space increased. There is obvious edema in ependymal region. The number of ependymal cells increased significantly: 101.9 ± 39.6 in the experimental group and 54.5 ± 3.4 in the sham group. There was a significant difference between the two groups (*P* < 0.0001) (Fig. 5d).

**Fig. 6:**
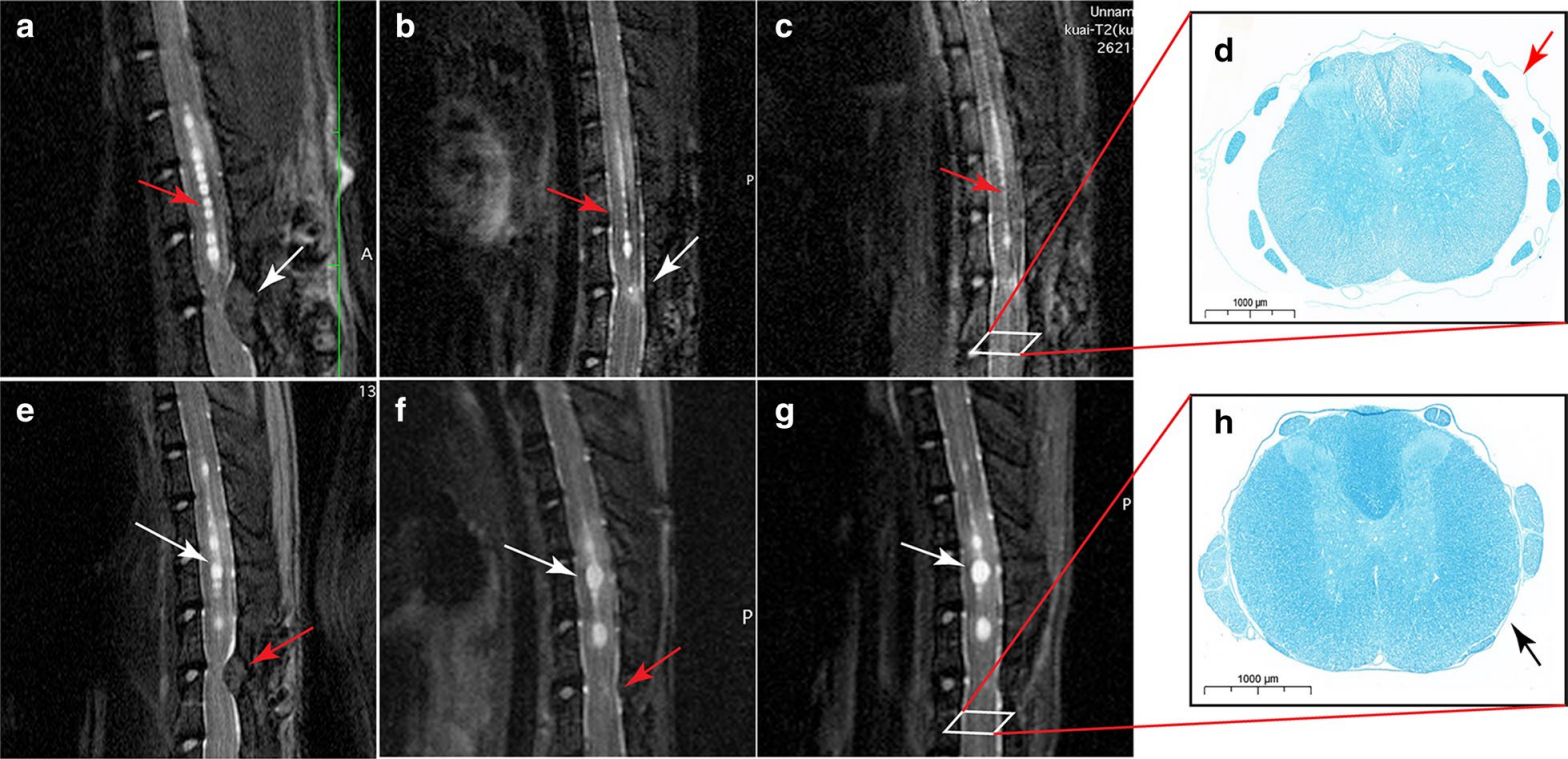
MRI and pathology after decompression. **a** Eight weeks after the operation to induce syringomyelia, the syrinx appeared in the center of the T10–12 spinal cord (red arrow). At the same time, cotton strips under the T13 lamina can be seen. **b** Four weeks after the cotton strip and T13 lamina were removed, CSF signals reappear in the subarachnoid space of the compression area (white arrow). The syrinx was significantly narrowed (red arrow). **c** Eight weeks after decompression, the syrinx was further reduced compared with that at 4 weeks. A series of MRI images from the syringomyelia operation to decompression. **e** Syringomyelia was clearly observed in the center of the spinal cord before decompression. **f** Four weeks after decompression, the T13 lamina and cotton strip disappeared. The number of syrinxes in the center of the spinal cord decreased, but the volume increased significantly. Many syrinxes appeared to merge into one. **g** Eight weeks after decompression, the volume of the syrinx increased (white arrow). **d**, **h** Eight weeks after decompression, the L1 spinal cord was transected and underwent Luxol fast blue (LFB) staining. **d** The image is of the spinal cord of the rat in which the syrinx is reduced after decompression. The space between the dura mater, arachnoid and spinal cord is very large, and the arachnoid does not adhere to the surface of the spinal cord (red arrow). In this way, CSF in the subarachnoid space will flow smoothly. **h** The image is of the spinal cord of the rat in which the syrinx continues to enlarge after decompression. The dura mater and arachnoid of the L1 spinal cord have multiple adhesions on the surface of the spinal cord (black arrow). This phenomenon will reduce CSF flow

Of the 8 rats that underwent the decompression operation, the syrinx gradually decreased in 4 rats and continued increasing in size in the other 4 rats during the follow-up period of 8 weeks. Due to tight adhesion and scar formation, decompression operations are difficult, but the 8 rats achieved good decompression (the standard is that CSF refilled between the dura mater and spinal cord) (Fig. 6b, c, f, g). On the following pathological sections, we found adhesion between the dura mater, arachnoid and spinal cord surface caudal (L1) to the compression site of the rats with continuously enlarging syringomyelia, and the subarachnoid became very narrow. However, the subarachnoid space at the same site of the rats with reduced syringomyelia was much wider, and the arachnoid did not adhere to the spinal cord. This finding suggested that after blocking CSF circulation for a long time (such as 2 months), the arachnoid caudal to the compression site may adhere to the spinal cord, and even if compression is relieved, the CSF circulation cannot return to normal. Notably, arachnoid adhesion was not found at the rostral compression site, probably because CSF above the compression was never completely blocked (Fig. 6d, h).

## Discussion

At present, the mechanism for syringomyelia formation is controversial and in the past few decades, many hypotheses have been proposed to explain it. Gardner [25] believed that CSF flows from the fourth ventricle into the central canal due to increased craniospinal pressure. However, later imaging and autopsy studies found that central canal enlargement rarely occurred between the syrinx and the fourth ventricle [26, 27]. Others believe that different types of syringomyelia, such as Chiari malformation, arachnoid inflammation, post-traumatic, tethered spinal cord, etc., have the same mechanism of syringogenesis [28]. These diseases all lead to changes in the fluid dynamics of CSF in the subarachnoid space, thus favoring the flow of CSF from the subarachnoid space into the central canal [29, 30]. Some studies have confirmed that CSF passes through the spinal cord to communicate between the subarachnoid space and central canal [13, 26, 31–33]. In our model, CSF flow was blocked due to extradural compression. Thus, turbulence increased and precipitated CSF flow to the central canal through the perivascular spaces (Virchow-Robin space), causing the central canal to expand.

In previous models that tried to induce syringomyelia by injecting kaolin solution or excitatory acid, syringomyelia was difficult to replicate [34, 35]. The heterogeneity of syringomyelia was relatively high. Kaolin and excitatory acid can kill neurons and induce inflammatory reactions and metabolic changes in the spinal cord [36]. In contrast to previous syringomyelia animal models, our model did not involve disruption of the dura mater to treat the spinal cord but only used extradural compression to block CSF flow. IBA1 is a marker of microglial cells, our result shows that syringomyelia induced by extradural compression will not cause obvious inflammatory reactions. MBP is a marker of oligodendrocytes and no change in the myelin sheath was obvious in our model. Similarly, there is no significant difference in GFAP between the two groups of rats. In the experimental group, compared with the sham group, the spinal cord had no obvious inflammatory reaction, and microglial cells showed no obvious proliferation. However, previous studies have shown that neuroinflammatory reactions may play a role in the progression of syringomyelia [37]. According to our results, it is impossible to conclude whether neuroinflammatory reactions participate in the formation of syringomyelia. This needs extensive study. These findings are conducive to maintaining the original microenvironment inside the spinal cord and are the basis for studying the pathological process of diseases using animal models. In addition, compared with previous spinal cord injury models, such as contusion, impact and excitatory amino acid injection, our rat model had fewer postoperative complications [38, 39]. Some rats had lower limb dyskinesia early after the operation but gradually recovered. Spinal cord injury caused by the compression was temporary and could spontaneously recover. No rat suffered bladder or intestinal dysfunction. Our method of establishing syringomyelia is safe and causes few complications. In addition, the lesions were all located in the central canal instead of the spinal cord parenchyma. This finding is very different from previous models of syringomyelia. CSF flow occurs in the central canal, subarachnoid space and ventricles, which are anatomically closely related. Syringomyelia is often accompanied by hydrocephalus, especially in Chiari malformation [40, 41]. However, no hydrocephalus occurred in rats in this study, and we do not have sufficient evidence to explain the reason. We suspect that this is related to the compression site located in the thoracolumbar segment and the CSF flow in the occipital regions not directly blocked.

Some studies have tried to compress the spinal cord with various substances (such as ﻿silicon and ﻿metal plates), but the pathological changes were mostly spinal cord injury, not syringomyelia [42, 43]. We used cotton strips to compress and obtained a high positive rate. Compared with rigid substances such as plastic pipe, cotton has good plasticity and can be reshaped according to the epidural space during the stuffing process so that the cotton is better fitted to the dura mater, and the subarachnoid space is more thoroughly blocked; furthermore, injury to the spinal cord is less likely. By quantitatively weighing cotton strips, we could accurately control the degree of compression on the spinal cord so that severe spinal cord injury caused by excessive compression could be avoided, as well as inadequate CSF flow blocking due to insufficient compression. This material is one of the key factors for successful modeling.

Interestingly, the locomotor function was partially damaged 1 week after the operation in our study, at which time the syringomyelia was not yet formed, and the locomotor function recovered naturally during the progression of syringomyelia. We speculate that the locomotor deficit we observed is caused by spinal cord compression, not syringomyelia. Clinically, locomotor impairment in patients with syringomyelia usually occurs later than sensory disturbance [44], which is related to a longer course of disease (maybe 10–20 years). The life span of rats is about 18 months, and our experimental observation time is 2 months. In addition, rats walk on four limbs. It may require longer observation times and the use of more accurate locomotor evaluation methods (such as gait analysis) for future research. In this study, we unified the overall experimental conditions to eliminate the interference from various confounding factors. Although all the rats received same weight of cotton strip (15 mg) and 82.5% of the rats showed syringomyelia, the volume and length of syringomyelia was still heterogeneous. However, in humans, the rate of progression of syringomyelia and the time of onset of symptoms vary greatly [44]. This is very confusing and more extensive research is needed in the future.

In our study, ependymal cells increased with the development of syringomyelia, which confirmed previous findings [13]. On the one hand, our results indicate that ependymal cells play an important role in the pathophysiological process of syrinx formation; on the other hand, ependymal cell proliferation may also be one of the secondary pathological results of syringomyelia. We found extensive edema in the subependymal region which has been described in other animal models of syringomyelia [19, 45]. In hydrocephalus, there are similar changes in the subependymal region, which suggests that syringomyelia and hydrocephalus are related in pathogenesis [19]. Edema and cell damage in the subependymal region may be closely related to secondary neuronal damage, thus causing sensory and motor disorders [22]. We counted ependymal cells in our study, but did not track the origin of these cells, which is very important for the pathophysiology of syringomyelia. The ependymal cells usually lie in a resting state [46], but they become activated and proliferative by spinal cord injury via Ngn2, Notch1 and BMP4 pathways [47, 48]. Then, the proliferated ependymal cells migrated into the injury sites to form a neurogenic niche and differentiate into various neural cells, including glial cell, neurons and oligodendrocytes. This process aims to repair the injured spinal cord. However, the repair is limited due to the formation of a glial scar and a deficiency of newly differentiated neurons [49], which may contribute to the central canal obliteration. According to Rodriguez el. al cell junction pathology of ependymal cells in lateral ventricle leads to the denudation of ependymal cells, resulting in a disruption of the ventricular zone and subventricular zone to trigger the onset of congenital hydrocephalus [50–54]. Based on our own observation and Rodriguez’s studies, we speculate that both of the proliferation and denudation of ependymal cells may have adverse effect on neurological repair and contribute to formation of syringomyelia and hydrocephalus. Therefore, appropriate manipulation of ependymal cells would be a promising therapeutic strategy to treat CSF disorder, and perhaps there is a common ependymal mechanism in the pathophysiology of syringomyelia and ventriculomegaly. Given that the ependymal region is the main aggregation site of spinal cord endogenous stem cells, future research on the effect of syringomyelia on endogenous stem cells should be performed.

Eight weeks after the compression was relieved, 4/8 of the syrinxes in the rats were reduced, while the other 4 increased. This result is consistent with the various clinical outcomes of syringomyelia after decompression. Pathological sections showed that the dura mater below the compression site of the rats without a reduced syrinx was adherent to the spinal cord surface, while the space over the spinal cord of the rats with a reduced syrinx was unobstructed. Previous clinical [55] and animal [34] studies have also proven that spinal arachnoiditis can lead to syringomyelia. This finding suggested that after CSF circulation is blocked for a long time (such as 2 months), the arachnoid caudal to the compression site may adhere to the spinal cord, and even if compression is relieved, the CSF circulation cannot return to normal. Our results also demonstrated that the obstruction of CSF flow in the subarachnoid space is an important factor in the formation of syringomyelia.

This study is an exploration of a new animal model of syringomyelia, which inevitably has some limitations. In human syringomyelia, neonates and infants account for a large proportion of cases [56]. However, our research used 8-week-old adult rats. The main consideration is that the adult rats are fully grown and the compressed cotton strip will not loosen during the observation period. However, it is still necessary to conduct syringomyelia induction experiments in younger rats. Our study focused on the pathogenesis and morphology of syringomyelia, which mimics human diseases, but further studies are needed on symptomatology and therapeutics. In addition, in human syringomyelia, symptoms usually occur after suffering from syringogenic factors for a long time, and the time dimension of formation and prognosis of syringomyelia is often as long as several decades [57]. In this study, the follow-up time of the rats was not sufficiently long (8 weeks). Also, when measuring the number of ependymal cells, we selected three levels of measurement for each rat, namely the largest part of syringomyelia and the 5 mm plane above and below it. However, the number of slices used for counting may still have been insufficient to eliminate bias. Further research is needed on the deeper mechanism of the formation and molecular pathological changes of syringomyelia.

## Conclusion

Chronic extradural compression of spinal cord can induce syringomyelia in rats. After compression was relieved, syringomyelia had different outcomes, similar to human disease. Compared with the noncanalicular syringomyelia model, our canalicular syringomyelia rat model can mimic the nontraumatic clinical situation more closely and provide a novel model for the study of disease mechanisms and therapies.

## Data Availability

The datasets supporting the conclusions of this article are available from the corresponding author on reasonable request.

## Abbreviations

MRI: Magnetic resonance imaging
BBB: Basso, Beattie and Bresnahan
IBA1: Ionized calcium binding adapter molecule 1
MBP: Myelin basic protein
IOD: Integrated optical density
AOD: Average optical density
FOV: Field of view
TR: Time of repetition
TE: Time of echo
CSF: Cerebrospinal fluid
